# Patients with cystic echinococcosis in the three national referral centers of Mongolia: A model for CE management assessment

**DOI:** 10.1371/journal.pntd.0006686

**Published:** 2018-08-09

**Authors:** Bolor Bold, Jan Hattendorf, Agiimaa Shagj, Bayar Tserendovdon, Tsendjav Ayushkhuu, Amgalan Luvsandorj, Jakob Zinsstag, Thomas Junghanss

**Affiliations:** 1 Department of Epidemiology, National Center for Zoonotic Disease, 20^th^ Khoroo, Songinokhairkhan district, Ulaanbaatar, Mongolia; 2 Epidemiology and Public Health Department, Swiss Tropical and Public Health Institute, Basle, Switzerland; 3 University of Basle, Basle, Switzerland; 4 Department of General Surgery, National Center for Maternal and Child Health, Bayangol district, Ulaanbaatar, Mongolia; 5 Department of General Surgery, First Central Hospital, Sukhbaatar district, Ulaanbaatar, Mongolia; 6 Section Clinical Tropical Medicine, Heidelberg University Hospital, Heidelberg, Germany; Negrar Hospital, ITALY

## Abstract

**Background:**

Mongolia is one of the endemic countries for cystic echinococcosis (CE), a zoonotic disease caused by the larval stage of *Echinococcus granulosus*. The goal of this study is to describe the current clinical management of CE in Mongolia, to capture the distribution of cyst stages of patients treated, and to contrast current practice with WHO-IWGE expert consensus.

**Methods:**

Hospital records of CE patients treated between 2008 and 2015 at the three state hospitals and fulfilling the inclusion criterion ‘discharge diagnosis CE’ (ICD 10 code B.67.0–67.9) were reviewed. Demographical, geographical, clinical and ultrasonography (US) data were extracted and analyzed. The annual surgical incidence was estimated. The digital copies of US cyst images were independently staged by three international experts following the WHO CE cyst classification to determine the proportions of patients which ideally would have been assigned to the WHO recommended treatment modalities surgery, percutaneous, medical (benzimidazole) treatment and watch & wait.

**Results:**

A total of 290 patient records fulfilled the inclusion criteria of the study. 45.7% of patients were below 15 years of age. 73.7% of CE cysts were located in abdominal organs, predominantly liver. US images of 84 patients were staged and assessed for interrater-agreement. The average raw agreement was 77.2%. Unweighted Kappa coefficient and weighted Kappa was 0.57 and 0.59, respectively. Mean proportions of images judged as stages CE1, CE2, CE3a, CE3b, CE4 and CL were 0.59, 0.01, 0.19, 0.08, 0.03 and 0.11, respectively. 40 cysts met the inclusion criteria of treatment modality analysis. The mean proportions of cases with a single cyst assigned to medical, percutaneous treatment, surgery and watch & wait were 52.5% (95% CI 42–65), 25.8% (95% CI 15–30), 5.1% (95% CI 0–10) and 3.3% (95% CI 0–10), respectively. 13.3% (95% CI 5–25) of cysts were staged as CL and therefore assigned to further diagnostic requirement.

**Conclusion:**

WHO CE cyst classification and WHO-IWGE expert consensus on clinical CE management is not implemented in Mongolia. This results in exclusively surgical treatment, an unnecessary high risk approach for the majority of patients who could receive medical, percutaneous treatment or observation (watch & wait). Introduction of WHO-IWGE expert consensus and training in ultrasound CE cyst staging would be highly beneficial for patients and the health care services.

## Author summary

Cystic Echinococcosis (CE) is a zoonotic disease, commonly known as dog tapeworm. The disease is distributed globally and predominantly affects rural populations with limited access to health care. Following the expert consensus of the WHO-Informal Working Group on Echinococcosis (WHO—IWGE) patients with uncomplicated cysts are assigned on the basis of WHO cyst classification to four treatment modalities: medical (benzimidazoles), percutaneous, surgical treatment, and ‘watch & wait’. In Mongolia, one third of the population practices nomadic farming. These populations are heavily affected by CE. However, cyst staging and WHO-IWGE recommendations are not implemented and patients referred to the three national treatment centres receive surgical treatment. This exposes a large proportion of patients to an unnecessary high risk approach who could be treated–depending on cyst stage—with benzimidazoles, percutaneously or observed (watch & wait). We reviewed the hospital records of patients with CE and admitted between 2008 and 2015 to the three national CE treatment centres, retrospectively staged the cysts and assigned the patients to the four WHO-IWGE recommended treatment modalities. We found a high proportion of patients in the study population who would have most likely benefitted from non-surgical treatment options.

## Introduction

Cystic echinococcosis (CE) is a zoonotic disease caused by the larval stage of *Echinococcus granulosus*. The life cycle of *E*. *granulosus* is maintained between the dog as the definitive host and various livestock as the intermediate host. Humans are considered as an aberrant intermediate host. Ingested larvae develop into cystic lesions, mostly in the liver and lung [1]. The disease is globally distributed including Central Asian countries and China [2–4]. The annual global burden of disease is estimated at 184,000 Disability Adjusted Life Years (DALYs) [5, 6]. Due to a large proportion of asymptomatic cases and underreporting the disease burden is widely underestimated. Pastoral communities in countries with limited resources bear the greatest burden [3, 5, 7].

In Mongolia, one-third of the population is engaged in extensive pastoral farming. The presence of a large livestock population accompanied by watchdogs, a big number of stray dogs, unregulated private slaughtering, and lack of health education are the main reasons for the heavy human CE exposure [8, 9]. Historically, due to strong public and veterinary action, the surgical cases decreased from 13% in 1946 to 2% in 1988 in the state hospital [10, 11]. After the Soviet Union collapsed in 1990, the veterinary and public health sectors weakened and many control programs for zoonotic disease, including CE, collapsed [12]. Currently, CE is not included in the national surveillance system, and official statistics are, therefore, unavailable [8, 9, 13]. There are few reports on the current transmission of CE in Mongolia including small scale serological surveys, hampered, however, by the sensitivity and specificity problems of serological CE testing [11, 14–18].

CE predominantly affects rural populations with very limited access to health care [19]. Diagnosis, cyst staging, treatment and follow-up depend on imaging [20, 21]. Ultrasonography, however, is only recently introduced in low and lower middle income countries (LICs and LMICs)[22–24]. Thus most endemic countries, including Mongolia, have not yet implemented the WHO Informal Working Group on Echinococcosis (WHO-IWGE) expert consensus [25, 26]. The core piece of the WHO-IWGE expert consensus is to triage on the basis of ultrasound-defined cyst stages into four groups: medical, percutaneous, surgical treatment (active cyst stage CE1 to CE3b) and ‘watch & wait’ (inactive cyst stages CE4 and CE5) [20, 27–30].

The goal of this study is to describe the current clinical management of CE in Mongolia, to capture the distribution of cyst stages retrospectively from stored ultrasound images, to critically contrast current practice in Mongolia with WHO-IWGE expert consensus and to suggest a LIC / LMICs-adapted implementation strategy for WHO-IWGE expert consensus.

## Materials and method

### Ethics statement

This work presented here was approved by the Medical Ethics committee of Mongolia (July 2014) and WHO ERC (27 Nov 2015).

### Study area and data collection

We reviewed the hospital records of patients diagnosed with CE and admitted between 2008 and 2015 to the three state hospitals conducting CE surgery in Mongolia: First Central Hospital (FCH), Third Central Hospital (TCH), National Center for Maternal and Child Health (NCMCH). Patients identified as probable CE cases in the peripheral or secondary hospitals are, as a rule, referred to the three state hospitals for confirmation and surgery. In the archives of the state hospitals, the medical records are chronologically stored in bundles of 150–200 reports. On the front page of each record the discharge diagnosis is recorded by the surgeon, based on histopathology which is done as a routine in the national hospitals. The ‘discharge diagnosis CE’ (ICD 10 code B.67.0–67.9) was the inclusion criteria for our study.

The following data were extracted from the patient records on data extraction sheets: demographic and geographic data (specified in Table 1), clinical symptoms and signs, ultrasonography (US) reports including number and size of cysts, US images, surgical reports and final diagnosis. US images (photos) were digitalized and stored. The data collected on data extraction sheets were double entered into a data base.

**Table 1:** Demography, socio-economic status (SES) and geographical data of the patients.

Patient characteristics	Frequency	
	n	%
**Age**		
<14	113	45.7
15–59	116	47.0
>60	18	7.3
***Total***	***247***	***100***
**Sex**		
Male	116	47.0
Female	131	53.0
***Total***	***247***	***100***
**Occupation**		
Paid employee	42	34.4
Self-employed	16	13.1
Employed in animal husbandry	12	9.8
Unemployed/retired/disabled	52	42.6
***Total***	122*	***100***
**Distance from secondary level hospital**		
<100 km	107	62.2
100 km-200 km	53	30.8
>200 km	12	6.9
***Total***	172**	***100***
**Type of home**		
Apartment	47	19.0
Yurt	200	81.0
***Total***	***247***	***100***

*- 60 pediatric cases and 65 cases without information on employment were excluded from calculating the percentage of occupation.

**- 60 cases from Ulaanbaatar city and 15 cases without an exact address were excluded from calculating the percentage of distance

### Data analysis

The demographic information including age, sex, occupation, type of home, and distance from health care was presented with the relevant percentage. The distance between secondary hospital (provincial general hospital) and current address of the patient was calculated. The levels of the clinical care in Mongolia are provided in Fig A in S1 Appendix.

The socio-economic status (SES) in the adult patients was stratified according to the labour force category of the National Statistical Office [31]. The current address of the patient was used to plot the geographical distribution by employing the ArcGIS 10.0 (ESRI 2011. ArcGIS Desktop: Release 10. Redlands, CA: Environmental Systems Research Institute). The annual surgical incidence was estimated based on the hospital records. The frequency of the clinical signs & symptoms was calculated. The size and number of CE cysts were presented in three different categories, <5 cm, 5–10 cm, and >10 cm and single cyst, 1–3 cysts, and multiple cysts, respectively.

The digital copies of US cyst images were independently reviewed by three international experts (reviewers) to estimate the frequency distribution of cyst stages according to WHO cyst classification (CL, CE1, CE2, CE3a, CE3b, CE4, CE5) in the patient population referred to the state hospitals for confirmatory assessment and treatment. The exercise was performed on the US images of abdominal lesions. The experts received no further information about the patients. Duplicate images of cysts were provided when available. If the reviewer felt unable to stage a cyst, e.g. because of poor quality of an image, the cyst was classified ‘not identifiable’ (NI). After the assessment, exclusions were made on the double images. If a double image of a cyst was assessed identical by the reviewers, we selected randomly one of the images. If a double image was assessed not identical by the reviewers, the one with fewer “NI” votes was selected to represent the cyst. Interrater-agreement was calculated using the Kappa statistic. Raw agreement was calculated as the proportion of cysts where the raters noted the same stage and kappa statistics for ordered categories (CL, CE1, CE2, CE3a, CE3b, CE4, CE5). We report both, unweighted and weighted kappa statistics with weights calculated as the square of the distance between the two ordinal groups. We judged agreement as poor if κ < 0.2; fair if 0.2 ≤ κ < 0.4; moderate if 0.4 ≤ κ < 0.6; substantial if 0.6 ≤ κ < 0.8; and good if κ ≥ 0.8. The estimates represent the arithmetic mean of the 3 pair-wise comparisons. “NI” judgements were considered as missing values which have been excluded pair-wise for raw agreement and kappa estimation.

Allocation of the cysts to WHO recommended treatment modalities was performed on the basis of cyst staging by the 3 reviewers and cyst size. Cases with images assessed as “NI” by all three reviewers were excluded; equally, cases with more than one cyst since completeness and ascertainment of US images was difficult retrospectively. The double images were excluded using the same algorithm as in the analysis of interrater-agreement. We calculated combinations of CE stage and cyst size for all three reviewer assessments separately and calculated the mean percentage. Based on this combination, the probability of assignment of cases to the treatment modalities as defined by the WHO-IWGE expert consensus were estimated for each case. Assignment of cysts to WHO recommended treatment modalities was performed as if all cysts were uncomplicated since the retrospective data did not allow to reliably differentiate complicated from uncomplicated cysts. Bootstrap resampling with 10,000 replicates was used for estimating the 95% confidence intervals for the treatment options. The analysis was conducted using the statistical package R v 3.4.0.

## Results

**Fig 1:**
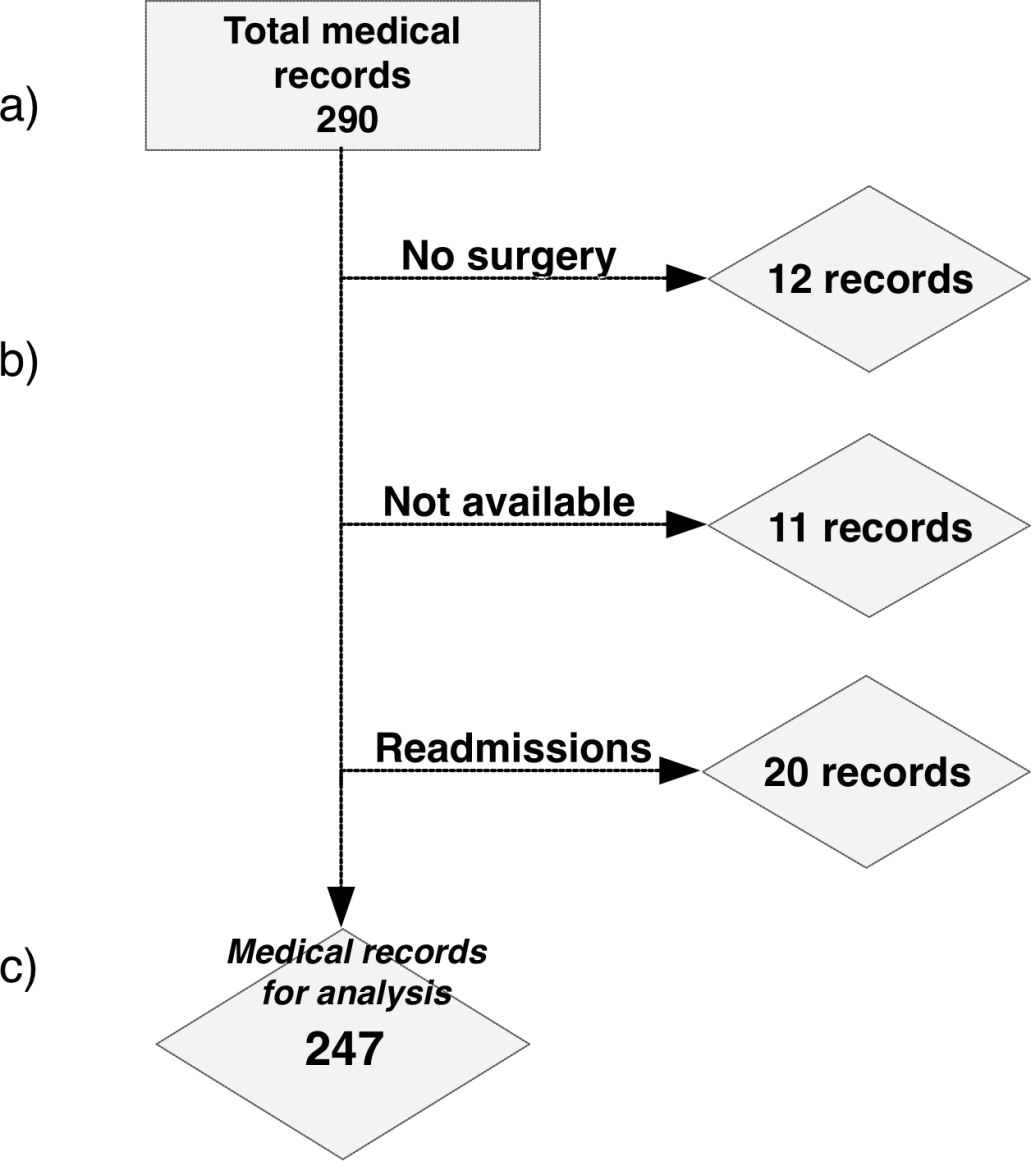
Selection of CE surgical case records. (a) 290 records (patients) in the 3 state hospitals in Mongolia fulfilled the inclusion criterion ‘discharge diagnosis CE’ (ICD 10 code B.67.0–67.9); (b) 12 records (patients) were excluded because the patients have not been operated on (4 surgeries postponed, 3 post-surgical conditions, 4 calcified cysts not needing surgery, 1 no information), 11 because of missing surgical information, 20 records belonged to 15 patients who were re-admitted once or twice; (c) 247 cases were analyzed.

A total of 290 medical records fulfilled the inclusion criterion ‘discharge diagnosis CE’ (ICD 10 code B.67.0–67.9) of the patients admitted to the three state hospitals between 2008 and 2015, 43 records were excluded. For details see Fig 1.

The demographic data are presented in Table 1.

During the period 2008–2015, the average annual CE surgical incidence per 100 000 was 1.06 (95% CI 0.7–1.4) based on the current data collection. On average 30.8 (95% CI 20.4–41.3) cases per annum underwent CE surgery in the central hospitals between 2008 and 2015 (Fig B in S1 Appendix).

**Fig 2:**
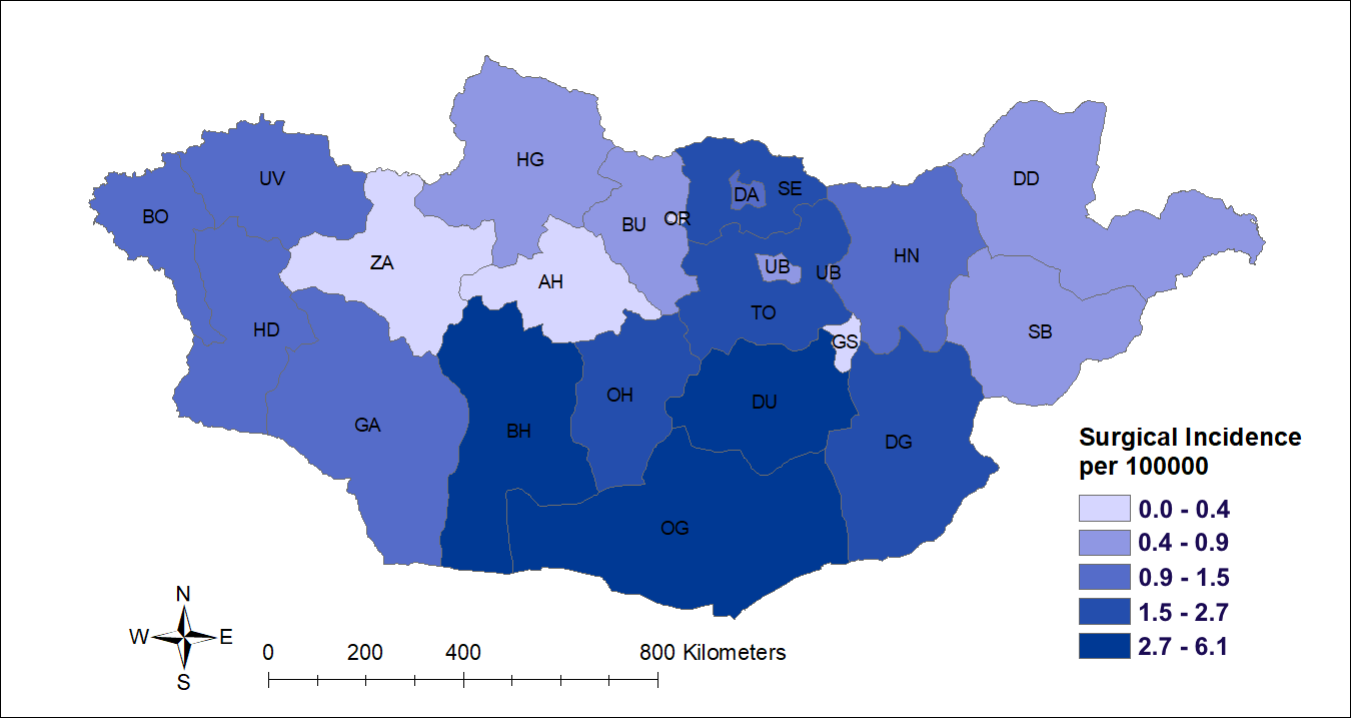
Average CE surgical incidence in each province for the period 2008–2015. Abbreviations: BO-Bayan-Olgii, UV-Uvs, HD-Khovd, ZA-Zavkhan, GA-Gobi-Altai, BH-Bayankhongor, OH-Ovorkhangai, AH- Arkhangai, HG-Khubsgul, BU-Bulgan, DA-Darkhan, SE-Selenge, ER- Orkhon, UB-Ulaanbaatar, DU-Dundgobi, OG-Omnogobi, TO- Tov, DG-Dornogobi, HN-Khentii, DD-Dornod, GS-Gobisumber., SB-Sukhbaatar.

The patients originated from 20 provinces corresponding to 95% (20/21) of all provinces (Fig 2). The southern provinces Omnogobi (OG), Dundgobi (DU), and Bayankhongor (BH) have the highest number of cases. Average CE surgical incidence for the survey period in these provinces was 2.7–6.1 per 100 000 inhabitants based on the medical records in the three state hospitals.

The frequency of symptoms & signs in CE patients at admission to the three state hospitals are provided in Fig C in S1 Appendix. The location of CE cysts of the surgically treated patients at the three state hospitals between 2008 and 2015 are provided in Fig D in S1 Appendix.

Preoperative ultrasound reports were available in 83.5% (152/182) of patients who had undergone abdominal surgery for CE. 26.3% (40/152) were explicitly recorded as hydatid cysts, 54.6% (83/152) as cystic lesions, and 19% (29/152) as other space occupying lesions. 99.3% (151/152) of the abdominal cases had the information on the cyst number in the US record. Among them, 78.1% (118/151) had single cysts, 20% (31/151) had 1–3 cysts and 1.3% (2/151) had more than 3 cysts. The information on size was available for 90.1% (137/152) of the abdominal cases. Among them, 48.9% (67/137) had cysts smaller than 5 cm, 35.0% (48/137) of 5–10 cm and 16.1% (22/137) were bigger than 10 cm.

**Fig 3:**
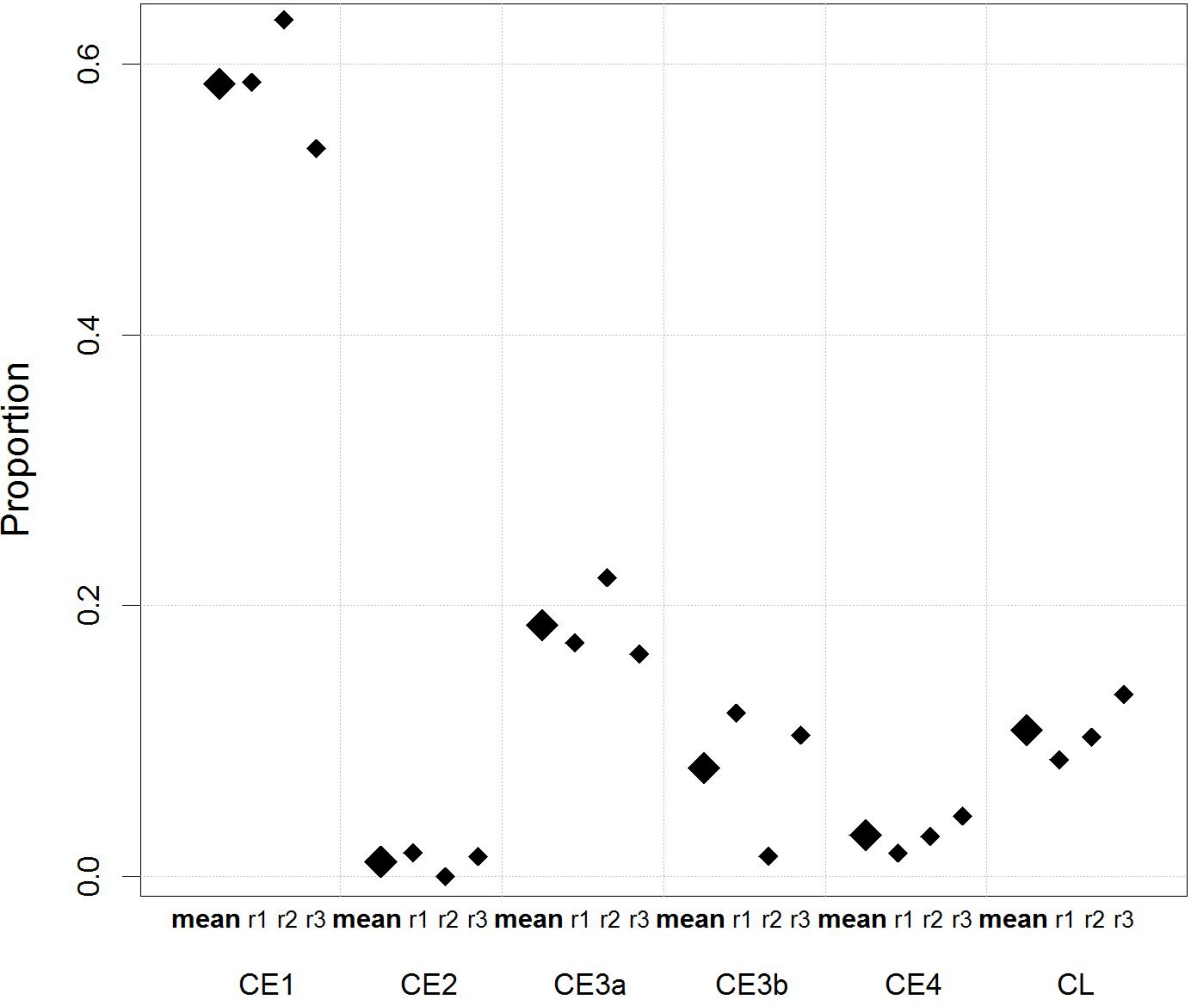
Distribution of CE cyst stages on the basis of the WHO CE cyst classification. The average proportions (big diamond) of CE cyst stages staged on the basis of the WHO CE cyst classification by the three experts. r1, r2, r3 are the proportions (small diamonds) of CE cyst stages classified by the expert 1, 2, and 3, respectively.

138 US imaging photos of 84 cases with liver and abdominal cysts were available for review and CE cyst staging based on the WHO CE cyst classification by three international reviewers. Following the inclusion / exclusion procedures described in the method section images of 84 unique cysts were assessed for interrater-agreement analysis. The raw agreement was 77.2%. We observed substantial agreement with an average unweighted Kappa coefficient of 0.57 and an average weighted Kappa coefficient of 0.59 (squared weights). The proportions of CE cyst stages are shown in Fig 3. Mean proportion for CE stages including CE1, CE2, CE3a, CE3b, CE4, and CL were 0.59, 0.01, 0.19, 0.08, 0.03 and 0.11, respectively. No CE5 cysts were identified.

**Fig 4:**
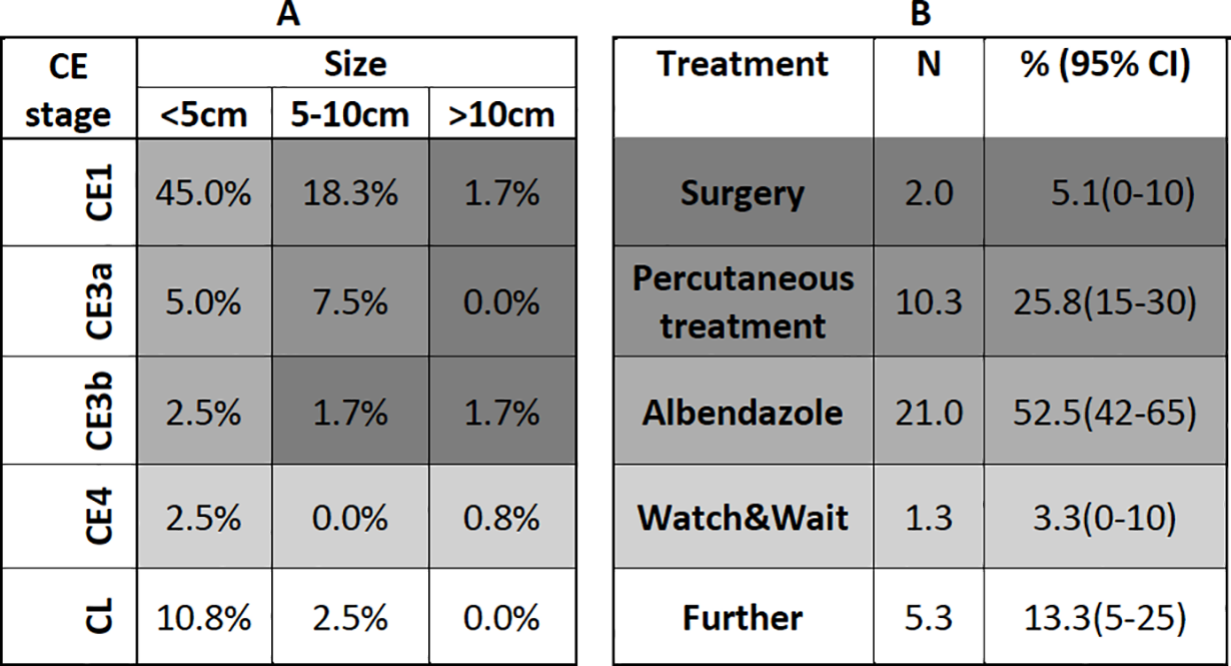
Assignment of CE cysts of the study population to WHO-IWGE recommended treatment modalities on the basis of retrospective cyst staging by three international experts. In uncomplicated CE cysts WHO-IWGE recommended assignment of patients to the four treatment modalities surgery, percutaneous methods, drug treatment (albendazole, mebendazole) and watch & wait is based on cysts stage and size (three groups). **A** Three size groups and the average percentages of WHO cysts stages in the study population as retrospectively staged by three international reviewers. Although there was a small percentage (1%) of CE2 identified by the three reviewers (Fig 3) this stage could not be included into the analysis because the information on cyst size was lacking. CE5 was excluded since none of the reviewers attributed one of the cysts presented for retrospective staging to CE5. **B** Based on the matrix in A, the probability of assignment of patients to the WHO-IWGE recommended treatment modalities is presented. The grey shading of the cells in A and B matrices correspond to each other. Cysts staged as CL would have needed further diagnostic work up.

Of the cysts staged by the three reviewers, 40 met the inclusion criteria for the treatment modality analysis (selection process see methodology section). The mean proportion of cases assigned to medical treatment, percutaneous treatment, surgery and watch & wait was calculated as 52.5% (95% CI 42–65), 25.8% (95% CI 15–30), 5.1% (95% CI 0–10) and 3.3% (95% CI 0–10), respectively. 13.3% (95% CI 5–25) of the cysts were staged as CL in need for further diagnostic work up (Fig 4).

## Discussion

To the best of our knowledge this is the first description of the clinical management of CE patients in LICs / LMICs with an attempt to critically contrast current national practice with the WHO-IWGE expert consensus [20] on the basis of interrater-agreement calculated from the retrospective cyst staging by three independent experts. Mongolia offers the unique opportunity for this exercise for two reasons. CE patients identified on the peripheral level of the health care services are exclusively referred for confirmation and treatment to the three state hospitals where patient records are carefully stored in archives with a discharge diagnosis on the front page of each patient file. The data of 290 patients with the ‘discharge diagnosis CE’ (ICD 10 code B.67.0–67.9) admitted between 2008 and 2015 were available to characterize the CE patients who currently receive treatment in Mongolia. Ultrasound photos of 46% (84/182) of patients with abdominal cysts were available for retrospective cyst staging on the basis of WHO CE cyst classification by three international experts. Based on the retrospective staging result and size, proportions of assignment to WHO-IWGE recommended treatment modalities were estimated.

The socio-demographic characteristics of the study population receiving treatment at the three state hospitals show some interesting features. Around 50% of the patients are below 15 years of age. In Kazakhstan and Kyrgyzstan, countries in the same region as Mongolia and with a similar pastoral lifestyle, also reported high proportion of children among the surgical cases [3]. Equal proportions of male and female patients suggest that, understandably, the infection risk is equal with CE transmitted in a food borne dog faecal-human oral cycle. Distance to the secondary hospital is a relevant factor for access to specialized health care in all LICs and most MICs [32, 33]. About 40% of the surgical patients had to travel at least 100 km to reach the secondary hospitals. Besides the geographic barrier other factors might contribute to underreporting but currently no data are available. 80% of the patients are living in a “yurt” which suggest that these patients are exposed to the combination of poor infrastructure, poor water and sanitation and frequent contact with watch-dogs and their faeces [34, 35].

A significant proportion of patients presented with non-specific symptoms, around 20% with abdominal pain corresponding to 29% of the patients with abdominal cysts. Around 16.6%, 8.5% and 6.9% of all patients had cough, chest pain and dyspnoea, respectively. This amounts to 50%, 32%, and 14%, respectively, of patients with lung cyst. These results are in line with the clinical experience that most patients present with non-specific symptoms and signs [36]. Fever, observed in 22.3% of all admissions, may, however, indicate that some patients had complication such as a cysto-biliary fistula with secondary bacterial infection of the cyst or cholangitis associated with biliary obstruction due to spillage of cyst content into the biliary tree; the latter also causes abdominal pain [37]. Similarly, patients with lung cysts may have had fever due to pneumonia following bronchial compression caused by expanding cysts or secondary bacterial infection of the residual cavity after the cyst content has been expectorated via a cysto-bronchial fistula. Both complications are associated with cough and dyspnoea [38]. Care should be exercised, however, to not over interpret retrospectively non-specific signs and symptoms such as fever, documented in medical records.

Most patients had only a single cyst in abdominal organs of which almost 50% had a diameter equal or smaller than 5 cm. This alone casts doubts on the indication for major surgery. If uncomplicated, these cysts would—following the WHO-IWGE expert consensus—either fall in the medical treatment group, if active (CE1 to CE3), or the watch & wait group, if inactive (CE4 and 5).

Only 40 reports of the 182 abdominal cysts examined by ultrasound spelled out “hydatid cyst” as the US diagnosis, without, however, mentioning accepted US criteria and without an attempt to stage with the WHO-IWGE or Gharbi classification.

The most exciting part of this data set was the unique opportunity to review and retrospectively stage 138 US imaging photos of 84 patients by three international experts to compare current practice in Mongolia with WHO recommended treatment modalities [20]. The distribution of WHO cyst stages retrospectively analysed from a subset of patients treated between 2008 and 2015 at the three state hospitals in Mongolia showed proportions of 0.59, 0.01, 0.19, 0.08, 0.03 and 0.11 for CE1, CE2, CE3a, CE3b, CE4, and CL, respectively (see Fig 3). Given the relatively high number of categories, the observed raw agreement of about 80% and Kappa coefficients of close to 0.6 indicate that the method appears to be generally valid but with room for improvement. A similar result was found in a recent study assessing interrater-agreement of the WHO cyst classification based on 2-D US images. The authors emphasized that an improvement can be achieved when non-static imaging (video recording etc.) is incorporated in future studies [39].

Assignment of patients to the WHO-IWGE recommended treatment modalities takes into account cyst size in addition to cyst stage. Merging the cyst sizes with the retrospective WHO cyst staging of the study population provides an insight into deviations of current practice in Mongolia from the WHO-IWGE expert consensus. Of particular significance for patients regarding unnecessary treatment risks and for the health care services with respect to avoidable treatment costs is the fact that 3.3% of abdominal cysts would have not warranted any treatment at all since they were already in an inactive stage (CE 4). Following the WHO-IWGE expert consensus they would have been submitted to the watch & wait approach with a very high probability of no need for further treatment [28, 29]. 52.5% of active abdominal cysts (CE1 to CE3) with a diameter of 5 cm or smaller would have been submitted to medical treatment following the WHO-IWGE expert consensus [20, 40]. This draws attention to the fact that availability of albendzole, the preferred benzimidazole for CE therapy, is hugely lacking in Mongolia. Currently, the price of albendazole is very high with approximately USD 2 per 400mg tablet. Albendazole is mostly sold in pharmacies in cities, which also limits access. Access to albendazole is a very much debated issue in the care for the neglected tropical disease echinococcosis in neglected populations [41]. In addition to albendazole cost, 10–15% of the hospital admission fee of public hospital is paid by the patients and the remaining cost is covered by the national health system in Mongolia [42]. However, there are many other costs including informal fees to clinicians and advanced diagnostics increasing the economic burden CE patients have to carry. Almost 26% of the patients assessed would have been allocated to percutaneous treatment (PT). PT options are generally underused and are not carried out in Mongolia for CE management due to a lack of trained personnel. Introducing PT into Mongolia should be considered because it carries less risks for the patients, reduces treatment cost and length of hospital stay. A study in Bulgaria showed similar deviations from best practices as recommended by WHO-IWGE expert consensus[43].

There are several limitations of the study. The data were extracted retrospectively from patient files. The fact that in Mongolia CE patients are being treated exclusively at the three state hospitals with high standards of documentation and archiving compensated partly for this shortcoming. Also, cyst staging was retrospectively performed on the basis of 2-D ultrasound photos. Our interrater-agreement of retrospective cyst staging, however, gives confidence that the staging result provides an acceptable estimate of the distribution of cyst stages seen at the three national hospitals. Identifying complicated cysts is difficult in retrospective studies. In some instances we could suspect that a cyst was complicated by combining various pieces of available retrospective information (e.g. fever, abdominal pain plus US cyst features suspicious of secondary bacterial infection of a cyst). This, however, is all too vague and would be an over interpretation of retrospective data. We thus did the analysis “as if” the cysts of the US images classified were uncomplicated. This may have resulted in an overestimate of the non-surgical treatment modalities.

In conclusion, our study demonstrates

1. Mongolia has a significant section of the population suffering from cystic echinococcosis and is in need of implementing a LMIC-adapted version of the WHO-IWGE expert consensus
2. Access to the privileged treatment centres, the three state hospitals, may be limited by geographic distance and economic resources
3. Ultrasonography is available, but diagnostic CE criteria seem not to be sufficiently well known and cyst staging is not performed
4. WHO-IWGE expert consensus for assigning patients to the four treatment options based on CE cyst staging and cyst size are not implemented resulting in surgical treatment of all CE patients and in an unnecessary high risk approach in patients who could be treated with albendazole or percutaneously or observed (watch & wait).

It is recommended

1. To systematically include CE into the disease surveillance system of Mongolia
2. To make ultrasonography available to the primary / secondary health care level and to train local, physicians in the WHO CE cyst staging. The Focused Assessment with Sonography for Echinococcosis (FASE), a short course for general practitioners, could be a model [26]. In 2016, a pilot training was conducted on WHO-CE cyst classification and staging for primary/secondary ultrasonography doctors by leading experts in the field.
3. To introduce and implement the WHO-IWGE expert consensus at all levels of the health care system to triage patients into the four WHO treatment modalities (a) medical treatment of small active cysts, (b) percutaneous treatment of larger active cysts (CE 1 and CE 3a), (c) surgery for complicated, very large cysts and cysts unresponsive to medical therapy, and (d) watch & wait for inactive cysts (CE4 and 5). Implementation at the primary and secondary health care levels saves transport cost, travel time for patients and allows a substantial proportion of patients to stay near or in their homes during treatment.
4. To apply our strategy of assessing CE clinical epidemiology and management in other CE endemic LICs / LMICs as a basis for planning.

## Supporting information

S1 Appendix (PDF)
