# Supplementary material for: Patients with cystic echinococcosis in the three national referral centers of Mongolia: A model for CE management assessment
Source: PLoS Negl Trop Dis. 2018 Aug 9;12(8):e0006686. doi: 10.1371/journal.pntd.0006686 (PMC6168150; doi:10.1371/journal.pntd.0006686)
Supplement: S1 Appendix — (PDF) [file pntd.0006686.s001.pdf]

S1 Appendix

Fig A. CE management provided at the three levels of health care in Mongolia

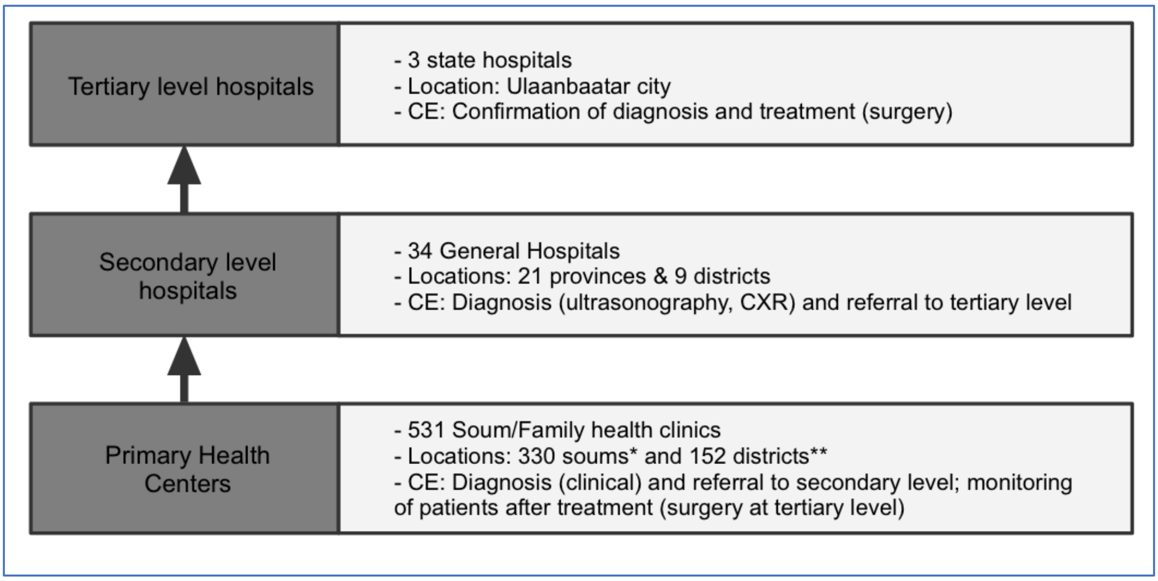

**- smaller administrative unit of province*

***- smaller administrative unit of district*

Fig B. CE surgical cases per year, 2008-2015

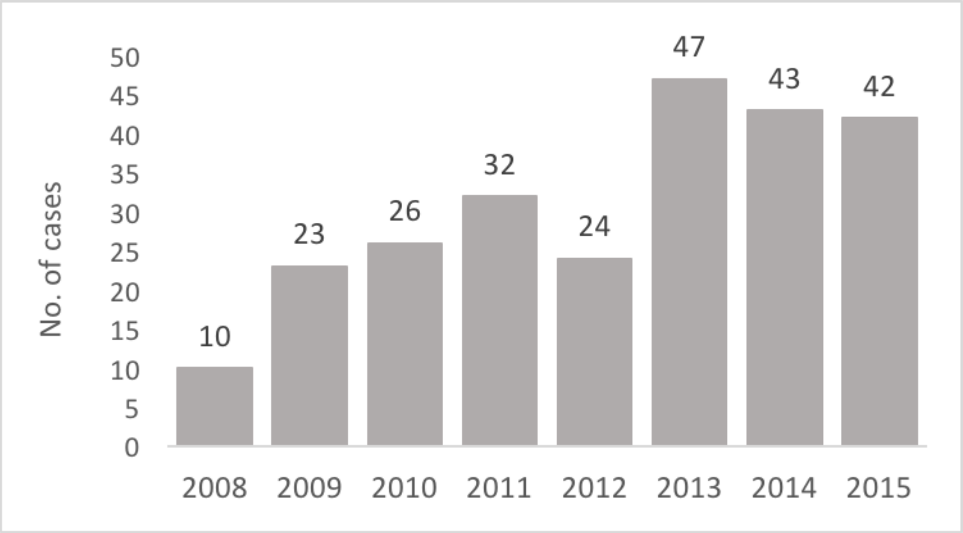

Fig C. Clinical symptoms and signs at admission to the three state hospitals

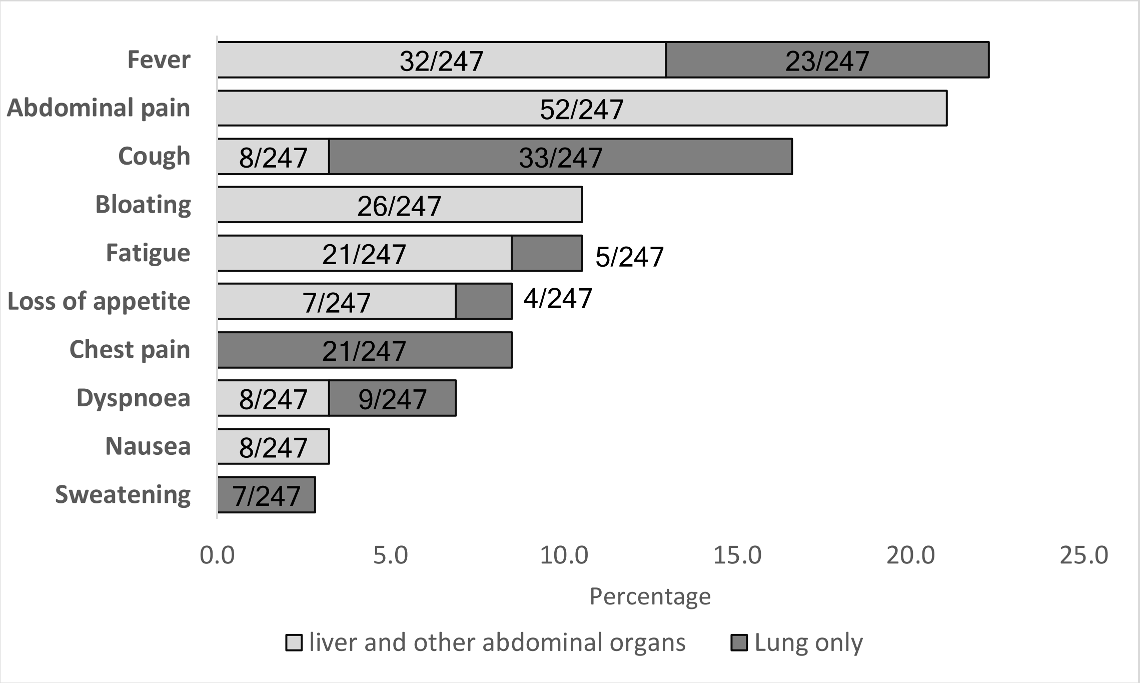

Numbers in bars represent $N_{reported}/N_{total}$.

Fig D. Locations of CE cysts of the surgically treated patients at the three state hospitals between 2008 and 2015

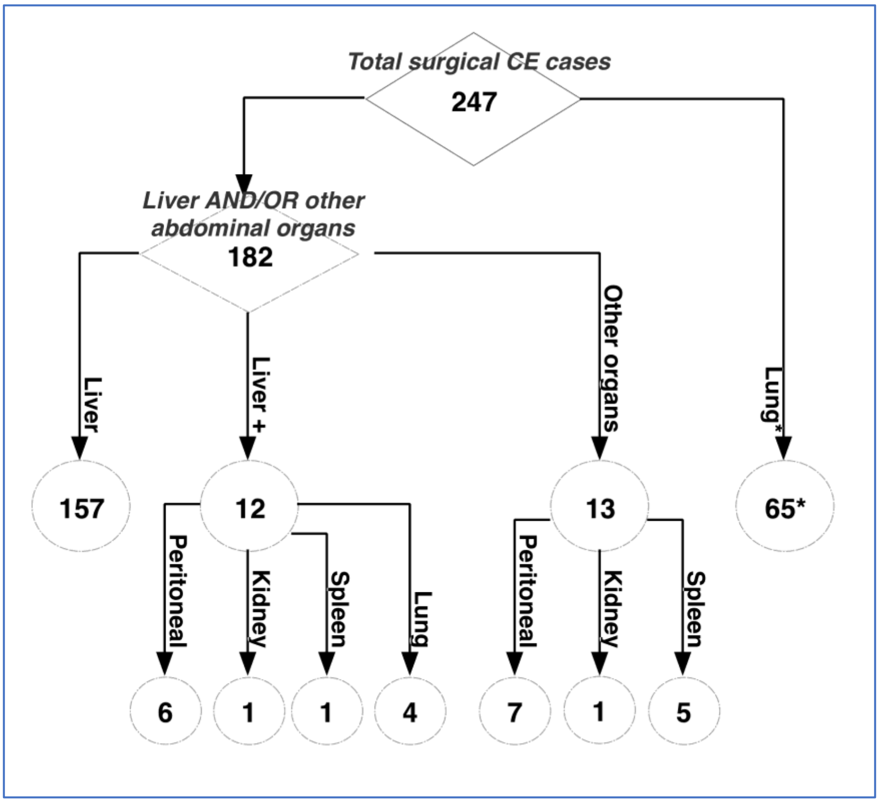

**Not analyzed for CE cyst stages (WHO CE cyst classification) due to unavailability of the X-ray films or other appropriate imaging*
